# Immunological Regulation of Fibrosis During Heart Failure: It Takes Two to Tango

**DOI:** 10.3390/biom15010058

**Published:** 2025-01-03

**Authors:** Vinay Kumar, Shyam S. Bansal

**Affiliations:** 1 Heart and Vascular Institute, Pennsylvania State University Hershey Medical Center, Hershey, PA 17033, USA; vkumar4@pennstatehealth.psu.edu; 2 Department of Medicine, Pennsylvania State University Hershey Medical Center, Hershey, PA 17033, USA; 3 Department of Cellular and Molecular Physiology, Pennsylvania State University Hershey Medical Center, Hershey, PA 17033, USA

**Keywords:** inflammation, monocytes and macrophages, T cells, myocardial infarction, ischemic heart failure

## Abstract

Immuno-fibrotic networks and their protein mediators, such as cytokines and chemokines, have increasingly been appreciated for their critical role in cardiac healing and fibrosis during cardiomyopathy. Immune activation, trafficking, and extravasation are tightly regulated to ensure a targeted and effective response against non-self antigens/pathogens while preserving tolerance towards self-antigens and coordinate fibrotic responses for efficient scar formation, a distinction that is severely compromised during chronic diseases. It is clear that immune cells are not only the critical regulators of post-infarct healing and scarring but are also the key players in regulating fibroblast activation during left-ventricular (LV) remodeling. Incomplete resolution coupled with sustained low-grade inflammation during dilated cardiomyopathy precipitates a “frustrated” immune cell response resulting in unconstrained pro-fibrotic and pro-hypertrophic signaling to induce maladaptive structural and functional changes in the myocardium. The aims of this review are to (i) briefly summarize the role of key immune cells that regulate wound healing during MI and fibrosis during LV remodeling; (ii) underscore phenotypic diversities in immune cells and their subsets to underscore their role in regulating fibrotic responses, and, last but not the least, (iii) highlight gaps in our understanding that restrict the translation of immuno-modulatory therapies from the preclinical models to heart failure patients.

## 1. Introduction

**Figure 1:**
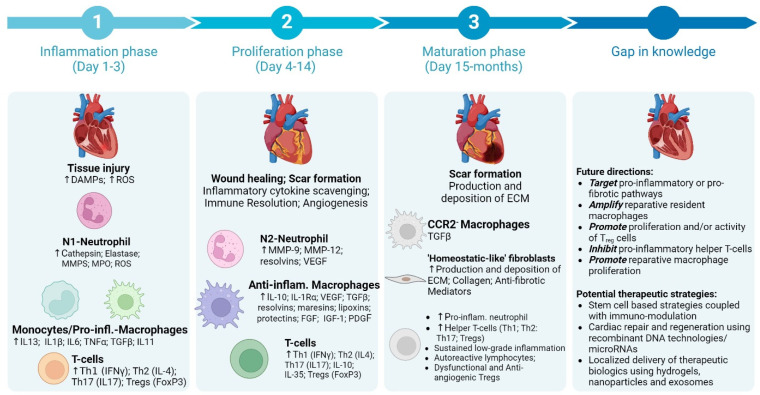
Schematic showing time course for the activation and trafficking of different immune cells into the heart post-MI and some key areas that need further exploration to clinically translate immuno-modulation strategies to ameliorate tissue fibrosis (Created in BioRender. McKenna, D. (2024); https://BioRender.com/d21o756, accessed on 18 December 2024).

**Figure 2:**
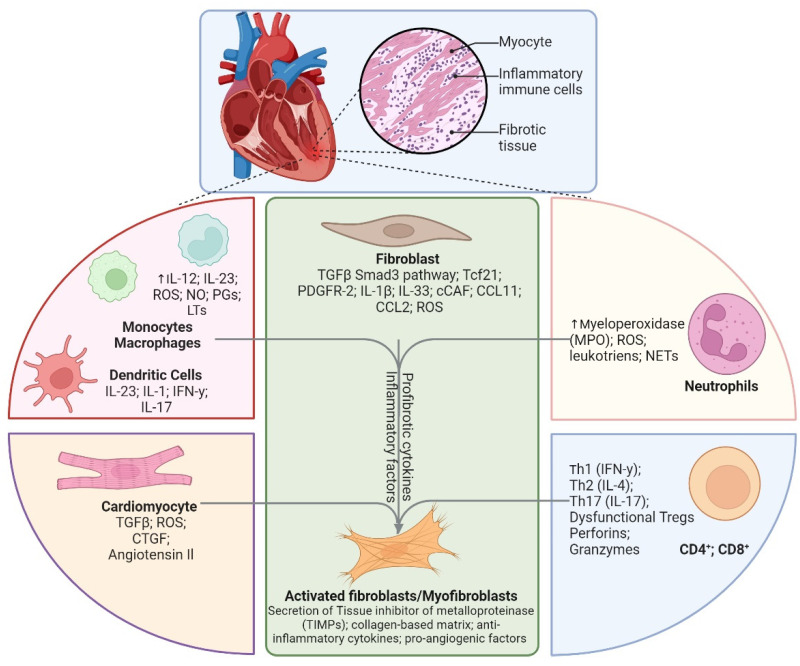
Schematic showing multi-factorial interactions between cardiomyocytes and different immune cells in mediating fibroblast activation (Created in BioRender. McKenna, D. (2024); https://BioRender.com/d21o756, accessed on 18 December 2024).

Cardiac fibroblasts are an integral part of the cardiac cell network and form a dynamic communication hub with other tissue cells such as the cardiomyocytes, endothelial cells, resident macrophages, and immune cells [1]. Fibroblasts are highly plastic and their phenotype is dynamically modulated by the micro-environment through direct cell–cell interactions with neighboring cells as well as by the cytokine/chemokine milieu to regulate extracellular matrix (ECM) deposition during homeostasis and disease states [1]. While ECM is needed to preserve tissue structure and facilitate paracrine and juxtracrine cell signaling, exaggerated ECM deposition as seen during HF, hypertension, diabetes obesity, or ageing can also result in tissue stiffening and decreased compliance, ultimately increasing cardiac load [1,2]. Cardiac fibroblasts are major determinants of tissue fibrosis post-injury [1], and diffused cardiac fibrosis is significantly associated with increased mortality in HF patients [3]. During chronic stress or sterile injury, cardiac fibroblasts differentiate into myofibroblasts which secrete matrix proteins and MMPs to spatio-temporally alter ECM structure and composition [4]. This process is initiated by tissue injury but is heavily regulated by inflammatory responses and activated immune cells either through direct cell–cell interactions or by way of released factors. Pre-clinical studies have shown that sequential activation of pro- and anti-inflammatory innate (such as neutrophils, monocytes, macrophages, and dendritic cells) and adaptive (such as T cells and B cells) immune cells is critical for fibroblast activation post-ischemic injury, such as in myocardial infarction (MI) [5,6,7]. Evolutionarily, this tight regulation is designed to sequentially clear dead cells followed by either the proliferation of tissue cells to re-establish homeostasis or fibroblast activation to fill empty spaces with collagen fibers [8]. Interactions between fibroblasts and different immune cells are central to the wound-healing cascades and tissue remodeling as seen during chronic diseases such as HF (Figure 1) and are mostly determined by the interacting immune cells (innate or adaptive) and their phenotype/transitory state (pro- vs. anti-inflammatory) [9]. Nevertheless, a delicate balance of immunological and fibrotic cascades is necessary to effect efficient wound healing and restrict pathological tissue remodeling post-injury, as shown in Figure 2. In this review, we briefly highlight some of the key interactions and signaling mechanisms between the immune cells and fibroblasts that determine fibrotic scar formation during ischemic and non-ischemic cardiomyopathy and underscore some gaps in our understanding to enable the development of potential therapeutic strategies.

## 2. Neutrophils in Fibrosis

Neutrophils are short-lived (10–12 h) innate immune cells of myeloid origin [10], and their circulating levels at 24 h post-hospitalization are directly associated with major adverse cardiovascular events in STEMI patients [11]. Preclinical studies have shown that neutrophil extravasation predominates during the initial hours after sterile injury, as in MI, to mediate necrotic-tissue clearance and produce pro-inflammatory cytokines and reactive oxygen species (Figure 2) [12]. They also facilitate recruitment of monocytes [6,13]. However, as the wound healing progresses, pro-inflammatory neutrophils give way to anti-inflammatory neutrophils for immune resolution and stable scar formation. While pro-inflammatory neutrophils produce S100A8/A9 and activate TLR4 signaling [11], IL-4 and TGF-β have been shown to characterize their anti-inflammatory phenotype [14]. Initial inflammatory responses by the neutrophils are critical for subsequent wound-healing cascades as depletion of neutrophils hampers phagocytic clearance of apoptotic cardiomyocytes, amplify myofibroblast proliferation and fibrosis, and exacerbate cardiac dysfunction [15]. Inflammatory protein effectors released by neutrophils, such as peptidylarginine deiminase 4 (PAD4) enzyme [16] or midkine [17], also directly enhance fibroblast activation and proliferation and overall fibrosis in the myocardium by promoting collagen synthesis and extracellular matrix deposition [4].

NETosis is one of the most potent processes by which neutrophils exert their inflammatory responses. Upon encountering an inflammatory stimulus (virus, bacteria or DAMPs), neutrophils release chromatin and granules enriched with antimicrobial proteins such as elastases, cathepsins, lipocalins, or myeloperoxide to form extracellular fibers called neutrophil extracellular traps (NETs) meant to bind and degrade virulent factors [18,19]. However, excessive NETosis, as seen in patients with myocarditis [17], during experimental autoimmune myocarditis (EAM) [17] and during ischemic HF [20] promotes inflammation and fibrosis. Targeting protein factors that mediate NETosis, such as midkine or PAD4, not only ameliorates inflammatory tissue damage but also blunt fibrosis and preserve systolic function during EAM [16,17]. Considering that fibroblasts are also known to release midkine when activated [21], it is possible that fibroblasts also regulate neutrophil NETosis, establishing a vicious cycle in which neutrophil and fibroblast activation pathways feed off each other. It is clear that neutrophils are obligatory for initiating an efficient wound-healing response and modulating fibroblast activity [15,22]; however, it is not clear how neutrophils dynamically change their phenotype during different stages of wound healing or how different neutrophil subsets mediate different functions dependent upon the severity and the type of the injury.

## 3. Monocytes and Macrophages in Fibrosis

Monocytes are functionally similar to neutrophils when it comes to their phagocytic potential but are long-lived, less cytotoxic, and utilize specific phenotypic markers for antigen/pathogen recognition, which renders them less prone to induce bystander tissue damage [23]. Monocytosis, like neutrophilia, is also strongly associated with MI prognosis and has been found to correlate with cardiac dysfunction, LV remodeling, and incidence of sudden cardiac death [24].

In rodents, monocytes with high Ly-6C (Ly-6C^hi^) are pro-inflammatory cells that patrol extravascular tissue spaces to phagocytose tissue/cellular debris and to transport them to the lymph nodes [25]. Cells that express low Ly6C (Ly6C^low^) are called patrolling non-classical monocytes as they continuously “patrol” the vasculature in an integrin-dependent manner [23]. They play a critical role in the clearance of damaged endothelial cells to maintain vascular integrity during a steady state [26]. In humans, classical and non-classical monocytes are defined as CD14^+^CD16^−^ and CD14^−^CD16^+^, respectively [21]. Both Ly-6C^low^ and -6C^hi^ subsets extravasate into the hearts post-injury and follow sequential recruitment (Figure 1). Pro-inflammatory phase I lasts for 1–3 days post-MI and is associated with the accumulation of Ly6C^hi^ monocytes in the myocardium to clear apoptotic cells [6]. Ly6C^hi^ monocytes are enriched with pro-inflammatory mediators (TNFα and IL1β) and have high proteinase activity to efficiently phagocytose dead cells and degrade extracellular matrix [27]. Anti-inflammatory phase II lasts from 4 to 7 days post-MI during which Ly6C^low^ monocytes infiltrate the myocardium to promote immune resolution and fibroblast activation [6]. Ly6C^low^ cells express TGF-β [28] and vascular endothelial growth factor (VEGF) [6], suggestive of their role in fibrotic and angiogenic processes. Accumulation of necrotic tissue areas upon Ly6C^hi^ depletion and decreased collagen deposition and neovascularization upon Ly6C^low^ depletion further support their differential functionalities post-MI [6].

Macrophages, although also of myeloid origin, exhibit significant phenotypic divergence from monocytes and can be of either embryonic (yolk-sac derived) or hematopoietic lineage [29]. While cardiac-resident macrophages (RMs) are embryonically derived, macrophages derived by the maturation of infiltrating monocytes are of hematopoietic lineage [30]. RMs are mostly maintained via local proliferation and are characterized by the expression of CD11b^low^F4/80^hi^CX3CR1^hi^ and not CCR2, a chemokine receptor used by monocytes for trafficking to the tissues [30]. Cardiac RMs constitutively transcribe genes required for DNA repair, coronary development, myogenesis, and epithelial–mesenchymal transition and express factors such as Pdgfc (platelet-derived growth factor C), Igf1 (insulin-like growth factor 1), Cyr61 (cysteine-rich angiogenic inducer 61), and Hbegf (heparin-binding epidermal-like growth factor) to conduct reparative functions in the myocardium [27]. PDGF released by RMs also helps maintain cardiac fibroblast as *PDGFRα^−/−^* mice have 50% fewer fibroblasts in the heart during homeostatic conditions [31]. RMs have also been shown to promote angiogenesis in mice with TAC-induced HF [32], which could be due to either increased endothelial cell proliferation or tip-cell fusion, as has been shown in other tissues such as the brain [33]. Due to their proximity to the cardiomyocytes, cardiac RMs are suitably positioned to rapidly respond to the DAMPs to initiate inflammatory responses by recruiting systemic myeloid and lymphoid immune cells [27]. Studies have shown that cardiac macrophages immediately after MI (2 d post-injury) are derived from the local proliferation of RMs which are slowly replaced by infiltrating monocytes and monocyte-derived macrophages (MDMs) by day 3–4 post-MI [27]. RNA sequencing of cardiac CD64^+^ monocytes and macrophages have shown 7 phenotypically distinct monocyte/macrophage subsets being present at 4 d post-ischemic injury [27]. Importantly, these subsets appear to respond to local cues encountered upon transmigration into the tissue and differentiate into diverse phenotypes to regulate neighboring cells, such as fibroblasts, endothelial cells, and immune cells. RM activation immediately after MI is necessary for adequate healing, angiogenesis, and fibrotic scar formation as depletion of CX3CR1+ RMs exacerbate peri-infarct fibrosis, LV remodeling, and cardiac dysfunction [32,34]. MDMs, on the other side, produce pro-inflammatory cytokines (IL-1β, TNFα) and chemokines (CCL2, CCL9, CxCl1, and Cxcl2) and are primed to activate NLRP3 inflammasomes suggesting an ontogenic shift replacing cardio-protective RMs with the pro-inflammatory MDMs [27,30]. CCR2^+^ MDMs are not only limited to the peri-infarct regions but also migrate to the areas remote from the ischemic sites [27], signifying their role in mediating phenotypic and structural changes in the remote zone as well, and their selective depletion before injury limits fibrotic scar expansion (akinetic area) and improves LV function [27]. Recent studies have underscored a key role of bromodomain-containing proteins (Brd) in MDM-dependent profibrotic effects. Conditional deletion of the transcriptional co-activator Brd4 in invading Cx3cr1^+^ MDMs ameliorates cardiac dysfunction in mice and markedly decreases fibroblast activation [35]. Brd4 binds to enhancer regions proximal to interleukin-1β (IL-1β) in Cx3cr1^+^ MDMs, which in turn activates RelA-dependent profibrotic mechanisms in fibroblasts [35]. MDMs have also been shown to promote fibroblast activator protein (FAP)/periostin (POSTN) signaling in fibroblasts within spatially defined niches via local release of IL-1β [36], and the deletion of either the IL-1 receptor on fibroblasts or the IL-1β ligand in MDMs reduces FAP/POSTN in fibroblasts, decreases myocardial fibrosis, and enhances cardiac function [36]. Brd4 is also a key regulator of fibroblast activation [37] suggesting a potent crosstalk mechanism in which Brd4 is able to mediate two-way cross-talk between the fibroblasts and MDMs by regulating local release of IL-1β. Among anti-fibrotic mechanisms, increased signaling through the CD74-MIF (macrophage migration inhibitory factor) axis, presumably via the anti-inflammatory macrophages, is a critical cross-talk mechanism between cardiac fibroblasts and MDMs [38].

Due to phenotypic plasticity and functional heterogeneity of various transitory states of monocytes and macrophages, it is not surprising that they modulate both pro- and anti-fibrotic responses by reacting to the tissue cytokine/chemokine milieu they encounter [27]. As a result, whether the mediators released by them, such as IL-1β, IFNγ, and TNFα, will promote myofibroblast differentiation to accentuate fibrosis or inhibit collagen and ECM synthesis is highly disease- and context-specific [39,40,41]. The exact cocktail of cytokines and chemokines which can fine-tune pro- vs. anti-fibrotic phenotypes of monocytes and macrophages in the injured myocardium is not yet known. If discovered, such cocktails can serve as effective therapeutics to modulate fibrotic processes for efficient wound healing and scar formation while minimizing interstitial fibrosis in the remote zone LV.

## 4. Dendritic Cells in Fibrosis

Dendritic cells (DCs) are phenotypically similar to macrophages due to their shared myeloid progenitor [42] but exhibit poor phagocytic activity and high antigen-presentation potential when compared to their myeloid counterparts [43]. Common DC progenitors, derived from a common myeloid progenitor, differentiate into plasmacytoid DCs (pDCs) or precursor DCs (pre-DCs) in the bone marrow (BM), which subsequently differentiates into conventional DCs (cDCs), cDC1 or cDC2 [44], expressing CD8α or CD4, respectively, in lymphoid tissues [42]. Once in non-lymphoid tissues, cDC1s acquire CD103 and XCR1 expression while cDC2s are characterized by CD11b and CD172α. Although, hearts have been shown to contain all of the above three (cDC1s, cDC2s, and pDCs) specialized DC populations, their distribution pattern has been shown to mimic RM distribution with the highest concentrations seen around the mitral and aortic valves [45]. During a steady state, dead and apoptotic cells are phagocytosed by the resident DCs, and the antigens are transported to the lymphoid tissues to induce T-cell anergy with and without their increased polarization into Tregs (iTregs) [46]. While CD103^+^ cDC1s are known to induce Treg polarization upon maturation, cDC2s promote pro-inflammatory polarization of T cells into Th1 and Th17 [47]. As a result, imbalanced cDC1 vs. cDC2 levels can disrupt immune homeostasis by either activating pro-inflammatory T cells or by reducing immune-suppressive Tregs [48].

DC levels increase in the heart as early as 2 d post-MI and reach their maximal levels by 7 d. While cDC2 levels peak at day 5, cDC1s and monocyte-derived DCs (MoDCs) are maximal by day 7 [45]. This sequential pattern is consistent with the fact that cDC2 maturation facilitates pro-inflammatory responses, whereas cDC1 activation is necessary to promote angiogenesis and induce tolerogenic Tregs to promote immune resolution [49]. Increased migration of mature cDCs from ischemic myocardium to the mediastinal lymph nodes [45] and their ability to effect antigen-presentation and activation of T cells in the lymphoid tissue [45] or in the heart [50] have also been reported. Plasmacytoid DCs (pDCs), on the other hand, are considered tolerogenic due to their ability to promote Treg polarization from effector T cells via the indoleamine 2,3-dioxygenase pathway. Similar to cDCs, pDCs are also recruited rapidly to the hearts post-MI from peripheral sources such as the spleen [45], presumably to carry processed antigens to the mediastinal lymph-nodes to induce Tregs and promote anti-inflammatory macrophage polarization in the hearts. This subsequently reduces profibrotic remodeling, promotes neovascularization and improves cardiac function at 28 d post-MI [51]. Considering that a phenotypic shift in pDCs, as seen during bleomycin-induced lung injury in mouse models, can also promote fibrosis by secreting IFNα and CXCL4 [52,53]; their role in promoting interstitial fibrosis during HF cannot be discounted.

Dendritic cells from the periphery, such as from BM or the spleen, can also move to the injury site and engage with T cells, macrophages, and fibroblasts, thereby modulating overall immune and fibrotic responses [50]. Like monocytes, peripheral recruitment of DCs, is also controlled by the IL-1β/IL-1R pathway as inhibition of IL-1R signaling (Figure 2), as in *IRAK1^−/−^* mice, results in decreased DC recruitment, inflammation, fibrosis, and hypertrophy [54]. Due to their ability to alter tolerogenic vs. immunogenic responses, DCs are potent modulators of the cardiac inflammatory milieu and fibrosis. Due to their phenotypic diversity, DCs can favor or inhibit fibroblast activation and subsequent collagen accumulation. Pro-inflammatory cytokines released by cDC2s can augment the recruitment and activation of other immune cells, such as macrophages, which are crucial in inducing fibrosis via the secretion of TGF-β, while anti-inflammatory cDC1s can inhibit fibroblast activation [55]. Although it is safe to presume an effect of cDC modulation on subsequent T-cell activation and Treg polarization, it is not clear to what extent observed protective effects are a direct result of DC depletion or are an indirect manifestation of an altered cytokine/chemokine milieu [45].

## 5. T Cells in Fibrosis

T cells are the orchestrators of adaptive immune responses and require activation of T-cell receptors (TCRs) by the cognate antigens presented by the APCs on their MHC-II proteins. Phagocytic uptake of apoptotic cardiomyocytes, processing of cardiac proteins, and subsequent emigration of APCs into the mediastinal lymph nodes [56] are all critical for antigen-specific T-cell activation and their subsequent trafficking to the ischemic myocardium. A recent study also showed an important role of fibroblasts in antigen uptake and presentation to the T cells during non-ischemic HF [57]. A rapidly changing local innate immune milieu during ischemic injury coupled with phenotypic heterogeneity of T cells necessitates tight control of regulatory vs. effector T-cell functions in a time-dependent context. Broadly, T cells can be divided into helper (Th) or cytotoxic (Tc) cells based on their expression of CD4 or CD8, respectively.

### 5.1. Helper T Cells and Fibrosis

During acute MI, circulating T cells have been found to be MHC-restricted and -enriched in specific T-cell clones, suggesting an antigenic T-cell response in patients [58]. Studies in mice with MI have further shown that Th-cell activation follows innate immune activation, with peak levels achieved by 3–5 days post-MI, which slowly decline to baseline levels by 2 weeks (Figure 1) [59,60]. Importantly, T-cell activation post-MI is required for efficient wound healing and fibrotic scar formation as CD4^−/−^ mice exhibit worsened ventricular dilation and disarrayed fibrosis with defective scar formation [61]. Considering the phenotypic diversity of Th cells, it is possible that different CD4^+^ T-cell subsets play different roles post-MI. It is known that while pro-inflammatory Th1 (IFNγ^+^) and Th17 (IL-17^+^) cells facilitate clearance of apoptotic cells by innate immune cells, anti-inflammatory Th2 (IL-4^+^) and Tregs (FoxP3^+^) play critical roles in fibrosis and angiogenesis during wound healing (Figure 2) [62]. Cytokines released by Th2 T cells, such as IL-4, IL-5, and IL-13, exert significant profibrotic activity in preclinical models of fibrosis in the lungs, skin, and the adipose tissues [63,64,65,66]. However, as opposed to discrete pro- vs. anti-inflammatory phases seen with monocytes, Th subsets do not exhibit sequential activation kinetics. Recent data have shown that both pro- (Th1 and Th17) and anti-inflammatory (Th2 and Tregs) Th cells increasingly infiltrate the heart post-MI, with the highest levels seen at ~3 d post-MI [60]. Nonetheless, systemic or cardiac predominance of pro-inflammatory Th cells during acute MI is apparent from several clinical [67,68] and pre-clinical studies [60,61]. It is thus possible that localized domains in the damaged myocardium are enriched in distinct cytokines and chemokines that polarize Th cells into different pro- and anti-inflammatory T-cell subsets that co-exist in different areas associated with either ongoing apoptotic cell clearance, fibrosis, or angiogenesis in the myocardium. Although cytokine/chemokine networks that regulate T-cell activation and recruitment during ischemic HF are unknown, studies in rodent models of non-ischemic HF suggest a critical role of the CXCR3/CXCL9/10 axis in T-cell recruitment [69]. Th cells, once in the tissue, can directly interact with fibroblasts to form α4-integrin-mediated junctions and augment TGFβ release to promote fibroblast differentiation into myofibroblasts [70]. The function of the Th cell in modulating fibroblast activity extends beyond its direct contact-dependent effects whereby effects originating from cytokines released by Th cells, such as IFNγ and IL-17, could also play an important role [71]. While IFNγ has been shown to promote cardiac fibrosis in rodent models of non-ischemic HF [70], IL-17 has been demonstrated to activate synovial fibroblasts [72]. Importantly, as opposed to MI where T cells are necessary for wound healing [61], CD4^+^ Th-cell depletion during the chronic phase (4 to 8 weeks post-MI) blunts LV dilatation, cardiac hypertrophy, and fibrosis [5,73]. These observations clearly establish that the effects of specific T-cell subsets are spatio-temporally altered during different phases of MI and HF. Considering these widespread effects of Th-cell subsets on wound healing and fibrosis, it is not surprising that blunted Th cells impact the overall inflammatory environment of the heart, thereby influencing fibroblast activity in several direct and indirect ways [9].

Tregs, a Th subset, are the regulators and suppressors of heightened immune activation during inflammatory diseases. They prevent overt immune activation against self-antigens and maintain immunological tolerance. Recent studies have shown that Tregs exhibit significant plasticity in their phenotype and function during MI and HF [73]. Tregs mediate wound healing by controlling pro- vs. anti-inflammatory immune responses and transition to the resolution phase from 4 to 10 days post-MI [73,74]. While Treg diminution during MI leads to excessive inflammatory immune responses, worse LV dilatation, and insufficient fibrotic scar formation [74], their adoptive transfer bestows significant protective effects by regulating immune activation during early phases of MI [74]. Tregs have been shown to decrease MMP-3 and α-SMA expression, resulting in suppressed transitioning of cardiac fibroblasts into activated myofibroblasts in a contact-dependent manner [74]. However, during chronic inflammation Tregs tend to lose their immune-suppressive potential during HF (Figure 2) and CAD [73,75,76]. Metabolic alterations [77], pro-inflammatory phenotypic shift [73], epigenetic changes [78], and/or polarization to Th1-like Tregs [79] are some of the cellular mechanisms known to be associated with loss in Treg competency. Reversing immune competency of Tregs either by depleting non-functional cells or by adoptively transferring immunologically competent Tregs suppresses pathological effector T cells, enhances vascularization, and blunts interstitial fibrosis and LV dilatation [73,74].

### 5.2. Cytotoxic T Cells and Fibrosis

CD8^+^ T-lymphocytes are cytotoxic T cells (Tc) and are known to induce cell death by releasing granzyme B (gzmB) and perforin enzymes [80]. They recently gained significant attention due to their ability to promote inflammatory responses by inducing damage to the already injured cardiomyocytes [81]. Systemic levels of GzmB, a potent serine protease released by Tc cells and capable of activating caspase mediated cell apoptosis, are predictive of increased mortality risk in patients with acute MI [80]. While clinical studies have shown increased Tc cells in circulation and endomyocardial biopsies in inflammatory DCM patients [82], preclinical studies have shown that activated CD8^+^ T lymphocytes infiltrate the ischemic myocardium as early as one day post-MI and alter cardiac healing by releasing pro-inflammatory cytokines and gzmB [80,81]. Increased gzm levels have also been reported in circulating Tc cells and cardiac tissue of inflammatory DCM patients [82]. Like CD4^+^ T cells, cardiac CD8^+^ T cells achieve highest levels at 3 d post-MI, which slowly decline to almost baseline levels by 14 days [80]. A second wave of increased CD8^+^ T-cell levels during ischemic heart failure (8 weeks post-MI) has also been reported [5]. While CD8^+^ T cells post-MI are phenotypically characterized by CD69 and CD107a in rodent hearts [80], increased CD57 expression on circulating CD8^+^ T cells is significantly associated with mortality within 6 months post-MI in patients [83]. Heightened expression of activation markers, such as CD69 and CD57, underscore pathological and significant effects of Tc cells during post-MI healing in both rodents and humans [84]. Importantly, CD8 T-cell activation appears to be antigen-specific, mediated by the clec9a^+^ cross-priming DCs [81]. Depletion of CD8^+^ T cells or CD8^+^ T cell-specific *gzm^−/−^* or DC-specific *clec9a^−/−^* ameliorate cardiac damage, remodeling, and fibrosis by decreasing cardiac gzmB levels or activation and transmigration of activated CD8^+^ T cells into the myocardium, respectively [80,81]. However, depletion of CD8α^+^ cells also results in delayed removal of necrotic tissue leading to poor fibrotic scar formation and increased cardiac rupture at 7 d post-MI [85]. Considering that CD8^+^ T cells do not mediate the transition from hypertrophy to HF, as has been seen in non-ischemic models of HF [86], it is safe to presume that protective effects of waned CD8^+^ T cells and defective fibrotic scar formation are due to toned-down inflammatory responses early after MI.

## 6. Gaps in Knowledge and Conclusions

Cross-talk between immune cells and fibroblasts post-MI is critical for efficient phagocytosis of apoptotic cardiomyocytes, neovascularization to regain tissue homeostasis, and fibrosis for scar formation to avoid cardiac rupture. Fine-tuning this cross-talk either by suppressing pro-inflammatory or promoting anti-inflammatory immune responses can thus also alter ECM to blunt LV remodeling and interstitial fibrosis. However, significant caution is necessary for such clinical strategies as even a slight immune imbalance to pro- vs. anti-inflammatory responses could result in paradoxical outcomes. While excessive pro-inflammatory immune cell activation could result in auto-immune reactions, under-activation of anti-inflammatory responses could result in inadequate wound healing and inefficient scar formation. Although our understanding of the inflammatory pathways that mediate LV remodeling and interstitial fibrosis during MI and HF has increased exponentially in recent years, several gaps in our understanding limit clinical translation of these findings. Some such limitations include (i) underlying signaling mechanisms that regulate immune cell cross-talk with fibroblasts are still poorly understood, (ii) different transitory states of immune cells and their functional roles are unknown, (iii) unavailability of experimental strategies to selectively deplete/inhibit one transitory state of immune cells vs. the other, (iv) poor understanding of temporal fluctuations in immune cell and fibroblast phenotypes, (v) underdeveloped human cardiac tissue or organoid models, and, last but not the least, (vi) inability of rodents and humanized mouse models to completely mimic disease and immune heterogeneity seen in humans, hampering effective translation into the clinics. Understanding immune mechanisms that regulate fibroblast phenotypes to mediate protective vs. pathological fibrosis and multi-factorial interactions that these cells exhibit with surrounding cellular networks is critical to devise anti-fibrotic therapeutic strategies.

## Data Availability

Not applicable.

## Abbreviations

MI: myocardial infarction; HF: heart failure; DAMPs: damage-associated molecular patterns, BM: bone marrow; CCL7: chemokine (C-C motif) ligand 7; CCR2/CCL2: C-C chemokine receptor type 2/chemokine (C-C motif) ligand 2; cDC: conventional dendritic cells; CX3CR1: CX3C chemokine receptor 1; DC: dendritic cells; IL-1β: interleukin 1β; Ly6C: lymphocyte antigen 6 complex; MDM: monocyte-derived macrophages; mLN: mediastinal lymph nodes; MyHCA: myosin heavy chain alpha; MoDC: monocyte-derived DCs, pDC: plasmacytoid dendritic cell; RM: resident macrophages; TCR: T-cell receptor; TGF: transforming growth factor; TNFα: tumor necrosis factor alpha; VEGF: vascular endothelial growth factor.
